# Extraction of Aloesin from *Aloe vera* Rind Using Alternative Green Solvents: Process Optimization and Biological Activity Assessment

**DOI:** 10.3390/biology10100951

**Published:** 2021-09-23

**Authors:** Mikel Añibarro-Ortega, José Pinela, Ana Ćirić, Elsa Lopes, Adriana K. Molina, Ricardo C. Calhelha, Marina Soković, Olga Ferreira, Isabel C. F. R. Ferreira, Lillian Barros

**Affiliations:** 1 Centro de Investigação de Montanha (CIMO), Instituto Politécnico de Bragança, Campus de Santa Apolónia, 5300-253 Bragança, Portugal; mikel@ipb.pt (M.A.-O.); amolina@ipb.pt (A.K.M.); calhelha@ipb.pt (R.C.C.); oferreira@ipb.pt (O.F.); iferreira@ipb.pt (I.C.F.R.F.); 2 Institute for Biological Research “Siniša Stanković”—National Institute of Republic of Serbia, University of Belgrade, Bulevar Despota Stefana 142, 11000 Belgrade, Serbia; rancic@ibiss.bg.ac.rs (A.Ć.); mris@ibiss.bg.ac.rs (M.S.); 3 aCourela do Alentejo, Garro Pinheiro de Baixo CCI3, Varche, 7350-422 Elvas, Portugal; info@acoureladoalentejo.com

**Keywords:** aloesin, *Aloe vera* green rind, process optimization, aqueous glycolic solvents, antioxidant activity, antimicrobial activity, cytotoxicity

## Abstract

**Simple Summary:**

Aloesin is a bioactive constituent of *Aloe* spp. used primarily in cosmetic products. Its recovery from plant materials is affected by several variables that can compromise the process yield and profitability, which is why it is necessary to determine the best processing conditions. This study describes the design and optimization of a method for extraction of aloesin from *Aloe vera* rind, a leaf part often discarded as a by-product, using the response surface methodology. The effect of the variables time, temperature, solvent composition, and solid/liquid ratio were investigated. Green organic solvents (ethanol, propylene glycol, and glycerol) were used in aqueous mixtures. Aqueous propylene glycol was found to be the most promising solvent for aloesin recovery and a linear increase in extraction yields was verified with the increase in solid/liquid ratio. To assess the bioactivity of the extracts, their ability to inhibit lipid peroxidation and the fungal and bacterial growth, as well as their cytotoxic potential, was tested in vitro. Overall, it was possible to determine the best extraction conditions for aloesin and to better understand the antioxidant and antimicrobial properties of the aloesin-rich extracts, which may be produced and used by the industrial sector.

**Abstract:**

Aloesin is an aromatic chromone with increasing applications in the cosmetic and health food industries. To optimize its extraction from the *Aloe vera* leaf rind, the independent variables time (10–210 min), temperature (25–95 °C) and organic solvent composition (0–100%, *w*/*w*) were combined in a central composite design coupled with response surface methodology. The solvents consisted of binary mixtures of water with ethanol, propylene glycol, or glycerol. The aloesin levels quantified in each extract were used as response for optimization. The theoretical models were fitted to the experimental data, statistically validated, and used to obtain the optimal extraction conditions. Then, a dose–response analysis of the solid/liquid ratio (*S*/*L*) was performed under the optimal conditions determined for each alcohol–water system and revealed that a linear improvement in extraction efficiency can be achieved by increasing the *S*/*L* ratio by up to 40 g/L. This analysis also allowed to experimentally validate the predictive models. Furthermore, the aloesin-rich extracts revealed antioxidant activity through thiobarbituric acid reactive substances (TBARS) formation inhibition, antimicrobial effects against bacterial and fungal strains, and no toxicity for PLP2 cells. Overall, this study provided optimal extraction conditions for the recovery of aloesin from *Aloe vera* rind through an eco-friendly extraction process and highlighted its bioactive potential.

## 1. Introduction

*Aloe vera* (*Aloe barbadensis* Mill.) is a succulent plant popularly recognized for its health-promoting effects and broad history of use in traditional medicine [1,2]. It is used worldwide mainly for the treatment of dermatological problems and the maintenance of healthy skin due to its healing, emollient, antioxidant, anti-inflammatory, antimicrobial, and depigmenting effects [3,4,5]. The inner leaf parenchyma is the most used plant part in the industry, while the photosynthetically active outer cortex (the thick epidermis with cuticle that corresponds to ~31% of the leaf weight) is often discarded as by-product with no commercial value [6,7]. Despite this, a high aloesin concentration is found in this green layer of the *Aloe vera* leaf [4].

The beneficial effects on the skin associated with *Aloe vera* have been linked to several bioactive compounds, including aloesin (also called aloeresin B) [8,9,10]. This *C*-glycosylated chromone is capable of inhibiting the tyrosinase activity, an enzyme responsible for catalysing the first stage of the conversion of tyrosine into melanin, and is therefore of high importance for the development of cosmetics [5,9]. It is used in patented products to protect the skin and hair from excess solar radiation [11] and to prevent skin aging [12]. This chromone also has potential as a functional food ingredient due to the beneficial effects in individuals in the pre-diabetic state or with metabolic syndrome [13]. In addition, some studies report the ability of aloesin to suppress the growth and metastasis of certain tumour cells and that it has no genotoxic activity [14,15].

The growing global cosmetics market has been looking for new bioactive ingredients of natural origin which are effective and absent of potential toxicity in order to meet the growing consumer expectations for products labelled as natural and produced in an eco-sustainable way [16]. In fact, both consumer and industry are avoiding products containing artificial additives such as benzoates, parabens, butylated hydroxyanisole (BHA), and butylated hydroxytoluene (BHT) [17,18,19]. An increasing number of companies has been replacing artificial molecules with natural ingredients in their products, which can also promote the valorisation of agro-industrial by-products, such as the recycling of *Aloe vera* rind into aloesin-rich extracts. Although the industry is being mobilized for a clean and circular economy, the existing processes for obtaining aloesin involve its conversion from aloeresin A (an ester of aloesin) extracted from the sap of *Aloe ferox* Mill. through hydrolytic processes employing non-eco-friendly inorganic acids, such as hydrochloric, sulphuric, nitric, and phosphoric acids [20]. Enzymes can also be used, but these were found less effective when applied to aloe bitter extract than when using purified aloeresin A [21].

The production of functional extracts involves several steps, including the identification of the compounds responsible for the bioactivity and the definition of the best conditions for their extraction. Although there are several standard solid–liquid extraction procedures for plant phytochemicals [22,23], they usually involve high consumption of organic solvents and energy, long processing time, and the possible degradation of target bioactive compounds [24,25,26]. Despite these disadvantages, these methods are usually easy to implement and execute at industrial scale level. However, the effectiveness of the solid–liquid extraction technique is affected by a number of factors that need to be optimized according to the intrinsic characteristics of the plant matrix and the solute. For this reason, it is crucial to select and optimize the processing factors and conditions case-by-case using suitable experimental designs and optimization tools such as the response surface methodology (RSM). There is also a need to explore alternative “green” solvents that address technical, economic, and environmental issues related to low-cost/low-energy demand production or that can be obtaining from industrial by-products, have reduced volatility and toxicity, and that are not cumulative neither in organisms nor in ecosystems [27].

Taking all these considerations into account, this study was carried out to optimize the extraction of aloesin from the *Aloe vera* green rind using green solvents and a central composite rotatable design (CCRD) coupled with RSM. Ethanol, propylene glycol (IUPAC: propane-1,2-diol), and glycerol (IUPAC: propane-1,2,3-triol) were chosen for the extractions given their potential to be used in different areas such as food, pharmaceuticals and cosmetics. In the particular case of the cosmetic industry, in addition to its general use as solvents, propylene glycol is also used as humectant, skin conditioning, and viscosity controlling, and glycerol as denaturant and humectant [28]. This suggests that both compounds could be part of a final formulation, thus eliminating the need for solvent separation from the extracted compounds. After optimization and validation of the predictive models, it was also intended to evaluate the antioxidant and antimicrobial activities, as well as the potential cytotoxicity of the aloesin-rich extracts obtained under optimized extraction conditions.

## 2. Materials and Methods

### 2.1. Plant Material

Freshly cut three-year-old *Aloe vera* leaves of organic production were supplied by “aCourela do Alentejo”, a company located in the parish of São Brás e São Lourenço, municipality of Elvas, Portugal. The green rind was separated from the inner fillet with a knife, lyophilized (FreeZone 4.5, Labconco, Kansas City, MO, USA) until a consistent weight was achieved, reduced to a fine powder (~20 mesh), and homogenised to obtain a representative sample that was kept at −20 °C until analysis.

### 2.2. Experimental Design for Extraction Process Optimization

A five-level CCRD coupled with RSM was implemented to optimize the extraction of aloesin from the *Aloe vera* rind. The coded and natural values of the independent variables *X*_1_ (time: *t*, min), *X*_2_ (temperature: *T*, °C), and *X*_3_ (organic solvent concentration: *S*, % ethanol, propylene glycol, or glycerol, *w*/*w*) are presented in Table 1. The ethanol–water, propylene glycol–water and glycerol–water mixtures were designated as EtOH-W, PG-W, and Gly-W systems, respectively. The 20 experimental runs of the CCRD design matrix shown in Table 1 were generated using Design-Expert software, Version 11 (Stat-Ease, Inc., Minneapolis, MN, USA) by entering the factor ranges in terms of alphas (*α* = 1.68). This rotatable design included 14 independent combinations and 6 replicated centre points. The axial points were chosen to allow rotatability, ensuring that the variance of the model prediction is constant at all points equidistant from the centre point of the design. The experimental runs were randomized to minimize the effects of unexpected variability.

### 2.3. Extraction Process

The extractions were performed in duplicate in a thermostated water bath (SW22, Julabo, Seelbach, Germany) using submersible magnetic stirrers (Cimarec, Thermo Scientific, San José, CA, USA) and sealed glass vessels to avoid solvent evaporation. Powdered samples (200 mg) were mixed with 20 mL of solvent and stirred at 500 rpm according to the experimental design matrix in Table 1, where different levels of *t* (10–210 min), *T* (25–95 °C), and *S* (0–100%) are combined. The solid/liquid ratio (*S*/*L*) was fixed at 10 g/L. After processing, the mixtures were centrifuged at 3000 rpm for 10 min and the supernatants were collected and kept at −80 °C until analysis.

### 2.4. Chromatographic Analysis of Aloesin

The extract solutions were five-fold diluted with water, filtered through 0.2 μm disposable filter disks, and analysed in a Dionex Ultimate 3000 HPLC system (Thermo Scientific, San Jose, CA, USA) as previously described [4,29]. Chromatographic separation was made in a Waters Spherisorb S3 ODS-2 C_18_ column (3 µm, 4.6 mm × 150 mm; Waters, Milford, MA, USA). Double online detection was carried out with a diode array detector (DAD) operating at 280 nm and a Linear Ion Trap (LTQ XL) mass spectrometer (MS, Thermo Finnigan, San Jose, CA, USA) equipped with an electrospray ionization (ESI) source. Aloesin was identified based on chromatographic data previously described [4] and quantified using a seven-level calibration curve (y = 3859.4x + 21770; *r*^2^ = 0.9996) constructed based on the UV-Vis signal of aloin (Alfa Aesar, Ward Hill, MA, USA) at concentrations ranging from 15.62 to 500 µg/mL. Data were processed using Xcalibur Software and the results were expressed as mg of aloesin per L of extract.

### 2.5. Extraction Process Modelling and Statistical Analysis

The aloesin content was the dependent (or response) variable used to optimize the extraction processes involving the three alcohol–water binary systems. Fitting procedures, coefficient estimates, and statistical analysis were performed using Design-Expert software as previously described [24,25]. Briefly, the variance analysis (ANOVA) was used to assess the significance of the generated polynomial model equations and of all the terms that make up these models, as well as the lack-of-fit. Only the statistically significant terms (*p* < 0.05) were considered in the development of the models (except those required to maintain hierarchy). The coefficient of determination (R^2^), the adjusted coefficient of determination (R^2^_adj_) and the adequate precision were used to estimate the adequacy of the polynomial model equations to the response. A non-significant (*p* > 0.05) lack-of-fit is desired so that the model can adequately describe the functional relationship between the three independent variables and the aloesin content. Design-Expert software was also used to generate the response surface graphs.

### 2.6. Dose–Response Analysis of the Solid/Liquid Ratio and Models Validation

After optimizing the experimental conditions for the variables *X*_1_, *X*_2_ and *X*_3_ involved in each of the three extraction systems, the solid–liquid ratio (*S*/*L*, g/L) was included as the fourth variable (*X*_4_) to be studied. For each solvent system, the powdered samples were processed under the optimal conditions defined in Table 2 at *S*/*L* ranging from 3 to 40 g/L. The dose–response effects as function of the *S*/*L* ratio increase were depicted using a general linear equation and the parametric slope value (*m*) was used for analysis. In turn, the model’s validation was performed by post-analysis verification in Design-Expert software (α = 0.05) with the experimental data obtained at 10 g/L.

### 2.7. Evaluation of Bioactive Properties

The three extracts obtained under the optimal extraction conditions of each alcohol–water system (Table 2) were evaluated for antioxidant, antimicrobial, and cytotoxic activities.

#### 2.7.1. Lipid Peroxidation Inhibition Capacity

A porcine brain cell solution (1:2, *w*/*v*; 0.1 mL) was incubated with 0.2 mL of extract solution at different concentrations plus 0.1 mL of FeSO_4_ (10 µM) and 0.1 mL of ascorbic acid (0.1 mM) at 37 °C for 1 h. Then, 0.5 mL of trichloroacetic acid (28% *w*/*v*) and 0.38 mL of thiobarbituric acid (TBA, 2%, *w*/*v*) were added and the mixture was heated at 80 °C for 20 min. After centrifugation at 3000× *g* for 10 min, the malondialdehyde (MDA)-TBA complexes formed in the supernatants were monitored at 532 nm (Specord 200 spectrophotometer, Analytik Jena, Jena, Germany) [30]. For each solvent system, a negative control was prepared with the respective extraction solvent. Trolox (Sigma-Aldrich, Saint Louis, MO, USA) was used as positive control. The results were expressed as EC_50_ values (µg/mL), i.e., extract solution concentration providing 50% of thiobarbituric acid reactive substances (TBARS) formation inhibition activity.

A one-way ANOVA was applied for assessing statistical differences between the results. The fulfilment of the ANOVA requirements, specifically the normal distribution of the residuals and the homogeneity of variance, was tested by means of Shapiro–Wilk’s and Levene’s tests, respectively. Data were compared using Tukey’s HSD test. The analysis was performed at a 5% significance level using SPSS Statistics software (IBM SPSS Statistics for Windows, Version 23.0, IBM Corp., Armonk, NY, USA).

#### 2.7.2. Antibacterial Activity

The extract solutions and extraction solvents were tested against the Gram (+) bacteria *Staphylococcus aureus* (ATCC 11632), *Staphylococcus epidermidis* (clinical isolate Ibis 2999), *Staphylococcus lugdunensis* (clinical isolate Ibis 2996), *Micrococcus flavus* (ATCC 10240), and *Listeria monocytogenes* (NCTC 7973), and the Gram (-) bacteria *Escherichia coli* (ATCC 25922), *Pseudomonas aeruginosa* (ATCC 27853), and *Salmonella enterica* subsp. *enterica* serovar Typhimurium (ATCC 13311), all obtained from the Mycological Laboratory, Department of Plant Physiology, Institute for Biological Research “Siniša Stanković”, University of Belgrade, Serbia. Minimum inhibitory concentrations (MIC), defined as was the lowest extract concentration (mg/mL) that inhibits the visible microbial growth (at the binocular microscope), were determined by the serial microdilution method and the rapid *p*-iodonitrotetrazolium violet (INT) colorimetric assay as previously described [31]. Minimal bactericidal concentrations (MBC) were determined by measuring the lowest concentration that yielded no growth and defined as the lowest concentrations (mg/mL) required to kill the original inoculum. Streptomycin was used as positive control.

#### 2.7.3. Antifungal Activity

The extract solutions and extraction solvents were tested against the fungi *Aspergillus flavus* (ATCC 9643), *Aspergillus niger* (ATCC 6275), *Penicillium funiculosum* (ATCC 36839), *Candida albicans* (clinical isolate Ibis 475/15), *Trichophyton mentagrophytes* (clinical isolate Ibis 2979/18), *Trichophyton* *tonsurans* (clinical isolate Ibis16/17), *Microsporum gypseum* (clinical isolate Ibis 3277/18), and *Microsporum canis* (clinical isolate Ibis 2990/18), all obtained from the Mycological Laboratory, Department of Plant Physiology, Institute for Biological Research “Siniša Stanković”, University of Belgrade, Serbia. The MIC and minimal fungicidal concentration (MFC) values (mg/mL) were determined as previously described [32]. Ketoconazole was used as positive control.

#### 2.7.4. Cytotoxic Activity

The cytotoxicity of the extract solutions was evaluated by sulforhodamine B (from Sigma-Aldrich) assay against PLP2 porcine liver primary cells as previously described [33]. Ellipticine was used as a positive control. The results were expressed in GI_50_ values (μg/mL), i.e., extract concentration providing 50% of cell growth inhibition.

## 3. Results and Discussion

Alternative green solvents have been increasingly exploited to extract bioactive phenolic compounds from plant materials, including propylene glycol and glycerol, which are generally applied in aqueous mixtures as co-solvents [34,35,36,37,38,39]. However, the extraction of these phytochemicals is also affected by other factors, such as processing time, temperature, and solid/liquid ratio, as well as by the intrinsic nature of the plant material. Therefore, in this study, the recovery of aloesin from *Aloe vera* green rind was optimized using RSM as optimization tool to assess the effects of relevant independent variables and possible interaction between them. The identification of the target *C*-glycosylated chromone (whose contents were used as the RSM response criterion) was performed based on chromatographic data (retention time, maximum absorption wavelength in the UV-Vis region, and mass spectrum) previously reported by Añibarro-Ortega et al. [4]. The glycolic solvents used in the extraction can be potentially used in the food, cosmetics, and pharmaceutical industries, in compliance with good manufacturing practice [28,40], representing an advantage over the commonly used organic solvents.

### 3.1. Experimental Data for Extraction Process Optimization

The experimental results obtained with the 20 runs of the five-level CCRD design matrix used to optimize the extraction of aloesin from *Aloe vera* rind are shown in Table 1. The aloesin concentrations ranged from 0 to 48 mg/L, 20.2 to 64 mg/L, and 21 to 57 mg/L with the tested EtOH-W, PG-water, and Gly-W mixtures, respectively. The lowest levels in the first two binary systems were achieved with run 14, which combined medium time and temperature conditions (110 min and 60 °C; 0 level) with a high solvent concentration (100% ethanol or PG (*w*/*w*); α = +1.68). In turn, the highest levels were achieved with runs 13 and 9, respectively, which combined medium time and temperature conditions (α = 0) with 0% ethanol (*w*/*w*), and a low processing time (α = −1.68) with a medium temperature and solvent concentration (0 level). In the Gly-W system, runs 7 and 8 resulted in the lowest yields thanks to the combination of medium-high temperatures and solvent concentrations (+1 level), conditions that also led to low yields in the PG-W system. In this binary system, the highest aloesin concentrations were reached with the axial points corresponding to runs 11 and 12. It should also be noted that run 13, whose conditions were exactly the same for the three solvent systems (since water was used as the extraction solvent; -1.68 level) yielded a similar aloesin content.

Regarding the six replicated centre points of the design matrix (Table 1), these resulted in mean values of approximately 33.9 ± 0.6 mg, 34 ± 3 mg, and 40 ± 2 mg per L, for the EtOH-water, PG-W, and Gly-W systems, respectively.

### 3.2. Models Fitting and Statistical Verification

The RMS is useful for the optimization of extraction processes involving one or more response variables, allowing the determination of factor interaction and optimal processing conditions with a reduced number of experimental trials, when compared with one-factor-at-a-time approaches [41]. To develop theoretical models capable of predicting the effects of the independent variables on a target response, it is necessary to assess the accuracy of their fitting to the experimental data. Therefore, the response values in Table 1 were fitted to a polynomial regression model using the Design-Expert software, considering only the significant terms (*p* < 0.05) in order to improve the models. The results of the regression analyses and analysis of variance (ANOVA) are shown in Table 2 and the polynomial model equations constructed for each extraction system in terms of coded values are presented in Equations (1)–(3).

For the EtOH-W system:

(1) $$\begin{array}{cc} Y_{\left(aloesin\right)}= & 33.9-2.9t-1.3T-14.1S-2.0t^{2}-7.2T^{2}-3.7S^{2}+0.92T^{3}+1.1tT\\ & \;+1.1tS+1.2TS+3.9t^{2}S+2.9tT^{2} \end{array}$$

For the PG-W system:

(2) $$\begin{array}{cc} Y_{\left(aloesin\right)}= & 33.0-9.0t-1.1T-8.2S+5.6t^{2}-0.8+1.8tS-3.9TS+5t^{2}S+7tT^{2}\\ & \;-12t^{2}T^{2} \end{array}$$

For the Gly-W system:

(3) $$\begin{array}{cc} Y_{\left(aloesin\right)}= & 40-3t-1T-6S+2.5t^{2}+5.7T^{2}+0.9tT+0.9tS-4t^{2}S+4tT^{2}\\ & \;-11t^{2}T^{2} \end{array}$$

In the equation models presented above, the parametric coefficients of each term illustrate the effect of the independent variable and their interactive effects. These represent the expected change in the response per unit change in factor value when the remaining factors are kept constant. The higher the parametric value, the more significant the weight of the variable is, and while a positive sign translates a synergistic effect on the extraction, a negative sign indicates an antagonism [42]. The complexity of the extraction trend is thus illustrated by the developed polynomial models. In each equation, the intercept corresponds to the overall average response of the 20 runs of the CCRD design; and these values were higher for the Gly-W system (Table 2).

As shown in Table 2, the three polynomial model equations presented high F-values (particularly the EtOH-W system), a non-significant lack-of-fit (*p* > 0.05), and an adequate precision greater than 17, thus suggesting that the models adequately describe the effects of the independent variables on the aloesin content [24]. The coefficients R^2^ and R^2^_adj_ were greater than 0.91 and 0.87 for Equations (1)–(3) and were in reasonable agreement with each other, indicating that the response variability can be explained by the variables involved in the extraction process [43]. An adequate precision of 17 indicated an adequate signal (Table 2) as it measures the signal-to-noise ratio. The developed polynomial models were thus statistically validated and used to navigate the design space. Although the model’s coefficients are empirical constants, they are useful for predicting the outcome of untested experimental extraction conditions.

Based on Table 2 and Equations (1)–(3), it can be observed that the extraction of aloesin from *Aloe vera* rind was affected by the three independent variables of the experimental design trough linear, quadratic, cubic, and interaction effects. In general, high-value parametric coefficients were observed in Equation (1) for the variable *S*, while *t* was particularly noted in Equation (2). In turn, high *T* coefficients were particularly noted in Equations (1) and (3). Strong negative interactions between *t*^2^ and *T*^2^ marked the PG-W and Gly-W systems, while *t*^2^ × *S* was detachable when EtOH-W mixtures were used. These results support the use of RSM for optimization, since the one-factor-at-a-time approaches do not evaluate interactions between independent variables.

### 3.3. Effect of the Extraction Parameters on Aloesin Content and Optimal Extraction Conditions

**Figure 1:**
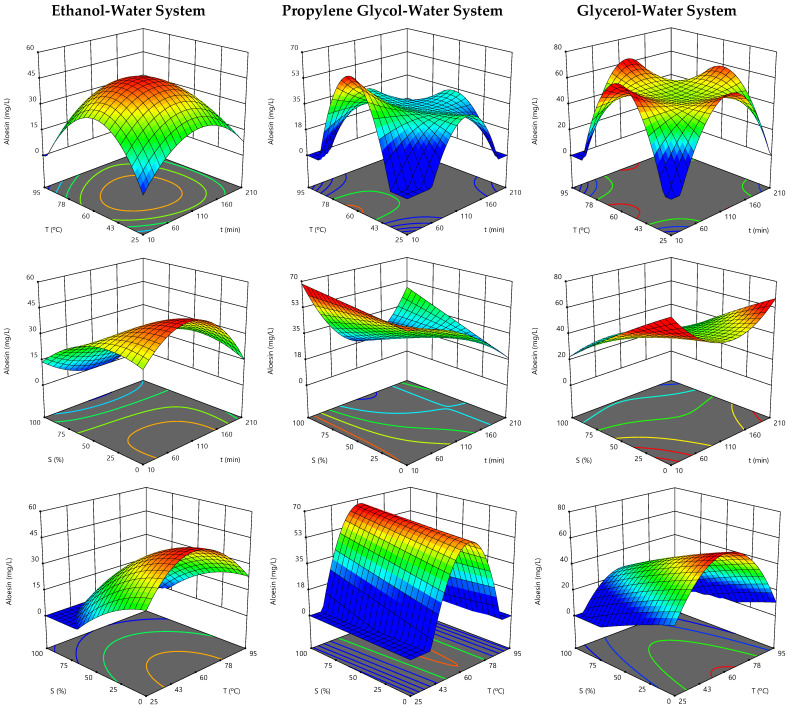
Response surface graphs for the effects of extraction time (*t*), temperature (*T*), and solvent (*S*) on the extraction of aloesin from *Aloe vera* rind as a function of the alcohol–water system. In each 3D graph, the excluded variable was positioned at its optimal value (Table 2).

**Figure 2:**
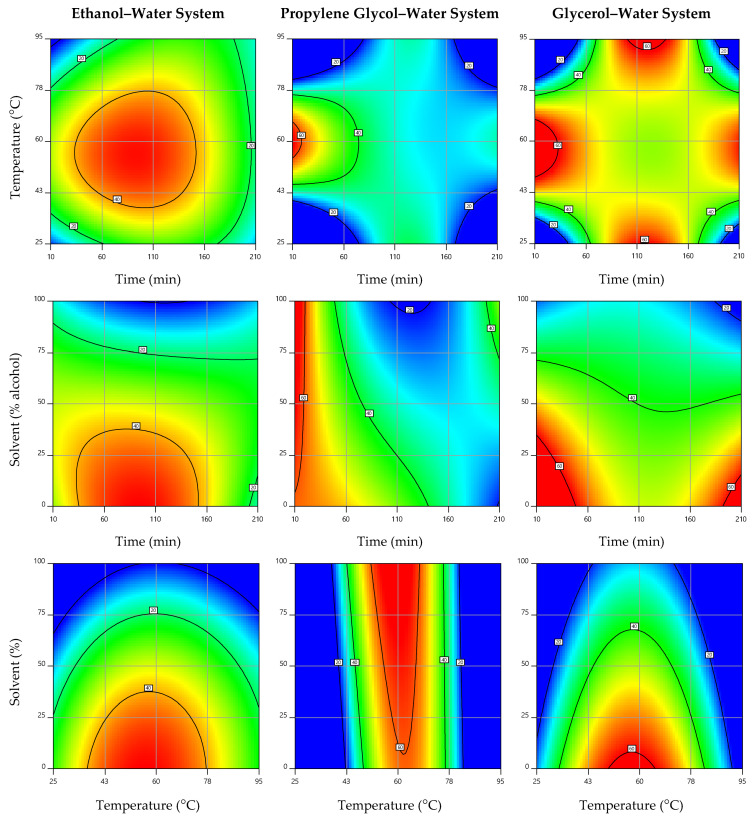
Contour plots showing the effects of the independent variables on the extraction of aloesin from *Aloe vera* rind as a function of the alcohol–water system. In each plot, the excluded variable was positioned at its optimal value (Table 2).

The response surface graphs constructed to illustrate the effect of the three independent variables involved in the extraction of aloesin from *Aloe vera* rind are presented in Figure 1, while the contour plots of the 3D graph projections are shown in Figure 2. In each representation, the excluded variable was positioned at its individual optimal value presented in Table 2. As observed in the different 2D and 3D graphs, as well as in Supplementary Material Figure S1, the tested extraction systems required a similar processing temperature; its increase up to 56–61 °C promoted the recovery of aloesin, which was then reduced with the temperature increase up to 95 °C, probably due to the thermal degradation of this high value-added bioactive chromone. Among the three systems, the one involving propylene glycol had the optimal temperature value (60.6 °C) slightly higher than those involving glycerol (56.8 °C) and water (55.9 °C) (Table 2). These results are in agreement with those of Kim et al. [44], who reported 60 °C as the best temperature for the extraction of aloesin from *Aloe vera* leaf gel.

For the variable time, the alternative systems of PG-W and Gly-W showed similar extraction trends and higher aloesin extraction yields for reduced processing times, namely 12 and 42 min, respectively (Table 2). On the other hand, the EtOH-W system showed an opposite trend, characterized by an increase in the recovery rate up to 93 min processing and a subsequent reduction for times up to 210 min. The optimum processing zone for this extraction system is perfectly illustrated by the red-coloured centre area of the response surface and its projection shown in Figure 1 and Figure 2, respectively, where the binary effects of temperature and time are combined. Thus, the PG-W system stood out as the most efficient for saving time, followed by Gly-W and then by EtOH-W.

The effects of the tested solvent binary mixtures were particularly noted in the EtOH-W and Gly-W systems (Figure 1 and Figure 2); the higher the alcohol concentration, the lower the extraction yield of the target compound. Therefore, 0% ethanol and 17.5% glycerol (*w*/*w*) were the most suitable solvents in the corresponding extraction systems, while 51.1% propylene glycol (*w*/*w*) was a promising binary mixture (Table 2). These optimal extraction mixtures can be related to the required time and temperature. In the EtOH-W system, the longer extraction time may be due to the use of water as the most suitable solvent. This was almost the same for the Gly-W system, where a low alcohol concentration is combined with a relatively short time. These two systems yielded 48 and 57 mg of aloesin/L, respectively (Table 2). Finally, the PG-W system allowed a shorter extraction time associated with a higher organic solvent consumption and a slightly higher temperature (probably to reduce the solvent viscosity). This system was the most promising in terms of performance, allowing to recover 63 mg of aloesin/L from the *Aloe vera* rind (Table 2). These results highlight the potential of alternative green solvents to extract aloesin from *Aloe vera* rind. Although beyond the scope of this work, the complex physicochemical interactions between the solvents and both the target solutes and the cell walls of the plant deserve further investigation in future works. Furthermore, while the glycolic mixtures can be applied directly to the formulation of various products (such as cosmetics), the aqueous extract offers the possibility of being dehydrated to obtain an aloesin-rich dry extract with a wide range of applications.

When testing ethanol–water mixtures to recover aloesin from *Aloe vera* leaf gel, Kim et al. [44] also found pure water as the most indicated solvent, while extraction times of 1 to 4 h did not induce significant effects. Thus, 1 h processing was sufficient. On the other hand, the maximum recovery yield of antioxidants (including both phenolic and non-phenolic compounds like carbohydrates) was achieved with 34% ethanol at 60 °C for 1 h. Overall, the organic solvent concentration was pointed out as the independent variable with the most influence in the extraction process, followed by temperature.

### 3.4. Models Validation and Effect of the Solid–Liquid Ratio

Low solvent consumption is an important requirement when designing new extraction methodologies. The volume should be sufficient only to dissolve the target compounds and promote mass transfer, maximizing the extraction yield with a minimum solvent consumption, thus making the process more sustainable and cost-effective, especially at industrial scale level. Therefore, after determining the processing conditions that maximize the extraction of aloesin from *Aloe vera* leaf rind with each solvent system (Table 2), these were applied to produce new extracts at *S*/*L* ratios ranging from 3 to 40 g/L in order to investigate the effect of this fourth variable and to evaluate the predictive accuracy of the polynomial models. The highest *S*/*L* ratio corresponded to the maximum *S*/*L* value that could be experimentally processed/stirred with the three tested extraction systems.

**Figure 3:**
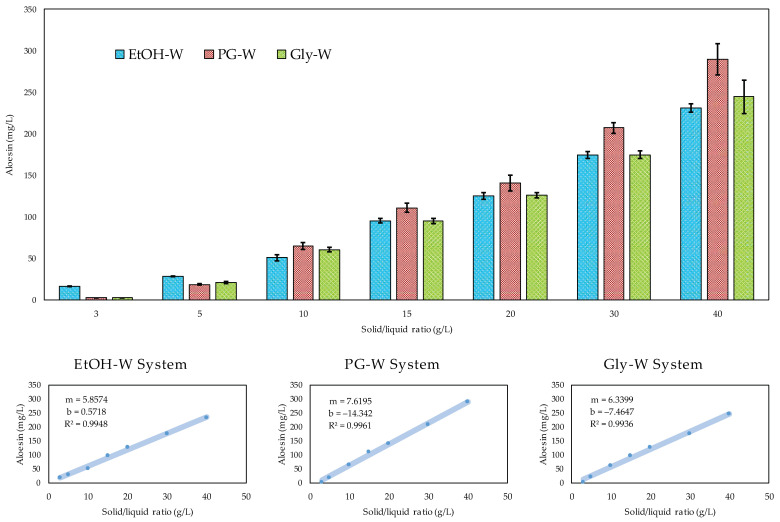
Dose–response effects of the solid/liquid ratio (*S*/*L*) on aloesin levels when processing the *Aloe vera* rind under the optimal conditions of time, temperature, and solvent concentration described in Table 2 for the three extraction systems. In the graphs at the bottom, the dots represent the experimental values and the lines show the extraction pattern predicted by the linear equation.

Figure 3 shows that the increase in the *S*/*L* ratio promoted a linear increase in the aloesin content. Those experimentally obtained at 10 g/L were in good agreement with the model-predicted values (Table 2), as confirmed by the post-analysis verification performed using the Design-Expert software (*α* = 0.05). The three extraction systems yielded 51 ± 4 mg, 65 ± 4 mg, and 61 ± 3 mg of aloesin per litre of solution, values that did not differ from the predicted 48 ± 1 mg, 63 ± 2 mg, and 57 ± 2 mg/L, respectively. The predictive capacity of the model equations was thus experimentally validated. For lower *S*/*L* ratios, EtOH-W was the most suitable extraction system, while above 10 g/L, the PG-W system stood out as the most promising to promote the mass transfer of aloesin. The dose–response effects as a function of the *S*/*L* increase showed a linear distribution and were depicted using a general linear equation with high R^2^ > 0.99 in all cases (Figure 3). Thus, based on the parametric slope value (*m*), it was confirmed that PG-W is in fact the most suitable system to promote mass transfer of aloesin mainly at higher *S*/*L* ratios, yielding 290 ± 19 mg/L when possessing at 40 g/L.

In the study by Kim et al. [44], the *S*/*L* ratio did not stand as a significant independent variable in the extraction process of aloesin from the *Aloe vera* leaf gel, probably because a lower range of values from approximately 17 to 33 g/L was analysed in the experimental design. Despite this, 22 g/L was the *S*/*L* ratio indicated for other response variables, namely aloin A and B and total phenolics. Thus, the expectation that the amount of solute in the liquid phase increases with the increase in *S*/*L* ratio was not observed by the authors.

### 3.5. Bioactive Properties of the Aloesin Extracts

The antioxidant activity of the aloesin-rich extracts obtained under the optimal extraction conditions of each solvent system (Table 2) was tested through the thiobarbituric acid reactive substances (TBARS) formation inhibition assay and the results are shown in Table 3. The lower the EC_50_ value, the higher the antioxidant activity. The extract obtained with the optimized EtOH-W system presented the lowest EC_50_ value (Table 3), and therefore the greatest TBARS formation inhibition capacity (*p* < 0.05), followed by the PG-W extract and finally by the Gly-W extract. This in vitro assay assesses the extract’s ability to inhibit the formation of malondialdehyde (MDA) and other reactive substances generated as a result of the lipid peroxidation of the used porcine brain cells. When the concentration of the extract does not prevent lipid peroxidation, the generated MDA forms a pink coloured complex with the thiobarbituric acid that is added to the mixture, which absorbs at a wavelength of 532 nm [45]. Therefore, the antioxidant mechanisms evaluated by TBARS offer information on the anti-peroxidative effect on lipids, which is important when developing shelf-stable cosmetic formulations containing aloesin or other bioactive agents.

In a previous study, Añibarro-Ortega et al. [4] described a lower IC_50_ value of 97 ± 3 µg/mL for a hydroethanolic *Aloe vera* rind extract, a value comparable to that of the fillet extract (87 ± 4 µg/mL). Nevertheless, it should be noted that these values are given in µg of extract while those of the present study are expressed in µg of plant material, which justifies the better antioxidant performance verified by Añibarro-Ortega and co-workers. According to Sun et al. [46], the antioxidant activity of *Aloe* species can be partially attributed to anthraquinones and related compounds, which display peroxyl radical scavenging activity and reducing power. Lucini et al. [47] also reported that aloesin, aloeresin A and aloesone are 5-methylchromones with strong radical scavenging activity and that the outer green rind is more bioactive than the inner parenchyma of the *Aloe* leaf. Therefore, although the present study focused only on one chromone, compounds other than aloesin may be present in the extracts and contribute to its bioactivity.

In food and cosmetic products, antimicrobial agents can help prevent the growth and proliferation of spoiling microorganisms and protect against microbial skin disorders and infections when in cosmetic formulations. Therefore, the aqueous and glycolic extracts obtained under optimal extraction conditions (Table 2) were tested against bacterial and fungal strains, some associated with skin problems and others of general occurrence. As shown in Table 3, the extract obtained with the optimized PG-W system was the most promising against most of the tested bacteria and fungi, with lower MIC (≤1.5 mg/mL) and MBC or MFC (≤2 mg/mL) values, respectively. *Salmonella enterica* serovar Typhimurium and *Listeria monocytogenes* were the most sensitive bacteria to the PG-W extract, with MIC (0.25 mg/mL) and MBC (0.5 mg/mL) values equal or slightly higher, respectively, to those of the antibiotic streptomycin used as positive control. In turn, *Candida albicans* was the most susceptible fungus to the PG-W extract, with 0.7 mg/mL and 1 mg/mL sufficient to inhibit and kill this opportunistic pathogenic yeast (Table 3). The Gly-W extract only stood out as the most promising against *Microsporum canis* (possibly due to the presence of compounds other than aloesin) and, in general, it was the least effective against the tested microorganisms, with no activity against some of them. Moreover, none of the solvents used to obtain the extracts showed antimicrobial activity, namely 51.1% propylene glycol (*w*/*w*) and 17.5% glycerol (*w*/*w*).

The antimicrobial activity of aloesin and *Aloe vera* extracts has been described by several authors. Hiruy et al. [48] showed that aloesin isolated from *Aloe monticola* Reynolds has strong activity against *Salmonella typhi*, *Shigella dysentery*, and *Staphylococcus aureus* (MIC of 0.01 mg/mL), as also against *Escherichia coli*, *Shigella boydii*, *S. flexneri*, and *S. soneii* (MIC of 0.025 mg/mL). In turn, 1 mg/mL was the MIC obtained against *Aspergillus niger*, *C. albicans*, *Penicillium funiculosum*, and *P. notatum*, a value much higher than that obtained for the positive control griseofulvin (0.014–0.016 mg/mL) and within the range of values obtained in the present study. However, a greater bioactivity would be expected from an isolated compound than from a plant extract. Interestingly, Añibarro-Ortega et al. [4] attributed a greater antifungal activity to the *Aloe vera* rind extract than to ketoconazole against *A. flavus*, *A. niger*, *P. funiculosum*, and *C. albicans*.

The potential toxicity to non-tumour primary PLP2 cells was also tested, but none of the three aloesin-rich extracts showed cytotoxicity at the tested concentrations (up to 400 µg/mL). Safety issues related to the *Aloe vera* rind extract cytotoxicity [4] and the aloesin genotoxicity [15] have already been excluded in previous studies.

## 4. Conclusions

This study is aligned with the concept of “green extraction” and societal challenges of the 21st century to obtain high quality and safe bioactive ingredients and protect the environment [49], as it describes the design of an eco-sustainable extraction process for the recovery of aloesin from an *Aloe vera* by-product based on the use of alternative non-toxic green solvents. The implemented experimental design combined three relevant independent variables (*t*, *T*, and *S*). The polynomial model equations were statistically valid for navigating the design space and predicting the processing conditions that maximize the recovery of this bioactive chromone. The highest aloesin yields (63 ± 2 mg/L) were achieved with 51.1% propylene glycol, which was the most efficient time-saving system. After experimental validation of the models, a linear improvement in the extraction rate was verified by increasing the *S*/*L* ratio up to 40 g/L (yielding 290 ± 19 mg aloesin/L with the PG-W system). In addition, the aloesin-rich extracts showed capacity to inhibit the formation of TBARS, antimicrobial effects mainly against *S. enterica* serovar Typhimurium, *L. monocytogenes*, and *C. albicans*, and no cytotoxicity to PLP2 cells. The bioactive aloesin-rich extracts developed in this study can be potentially used in foods, cosmetics, and other products, in compliance with good manufacturing practices.

## Figures and Tables

**Table 1:** Aloesin concentrations obtained experimentally under the extraction conditions defined by the CCRD design matrix as a function of the applied alcohol–water system. The levels of the independent variables are presented in the actual and coded (in brackets) form.

Run	Experimental CCRD Design Matrix			Aloesin Content (mg/L)		
	*X*_1_: *t* (min)	*X*_2_: *T* (°C)	*X*_3_: *S* (%)	EtOH-W System	PG-W System	Gly-W System
1	51 (−1)	39 (−1)	20 (−1)	35 ± 1	30.0 ± 0.6	46 ± 4
2	170 (+1)	39 (−1)	20 (−1)	30.2 ± 0.9	24 ± 2	45 ± 2
3	51 (−1)	81 (+1)	20 (−1)	30 ± 1	38.7 ± 0.9	39.7 ± 0.8
4	170 (+1)	81 (+1)	20 (−1)	29.8 ± 0.8	31 ± 2	44.4 ± 0.6
5	51 (−1)	39 (−1)	80 (+1)	9.5 ± 0.1	28± 4	27 ± 2
6	170 (+1)	39 (−1)	80 (+1)	9.3 ± 0.1	28.1 ± 0.6	31.3 ± 0.7
7	51 (−1)	81 (+1)	80 (+1)	9.5 ± 0.1	20.6 ± 0.3	21 ± 3
8	170 (+1)	81 (+1)	80 (+1)	13.85 ± 0.07	20.8 ± 0.1	21 ± 1
9	10 (−1.68)	60 (0)	50 (0)	33.4 ± 0.9	64 ± 1	52 ± 3
10	210 (+1.68)	60 (0)	50 (0)	23 ± 1	33.7 ± 0.4	42 ± 1
11	110 (0)	25 (−1.68)	50 (0)	11.1 ± 0.2	37 ± 1	57 ± 3
12	110 (0)	95 (+1.68)	50 (0)	16.5 ± 0.2	33 ± 1	55.2 ± 0.7
13	110 (0)	60 (0)	0 (−1.68)	48 ± 1	47.7 ± 0.3	47.9 ± 0.7
14	110 (0)	60 (0)	100 (+1.68)	tr	20.2 ± 0.8	28.29 ± 0.01
15	110 (0)	60 (0)	50 (0)	35 ± 1	35 ± 3	40 ± 2
16	110 (0)	60 (0)	50 (0)	33 ± 1	29.0 ± 0.1	39 ± 4
17	110 (0)	60 (0)	50 (0)	33 ± 2	37 ± 1	43 ± 4
18	110 (0)	60 (0)	50 (0)	34 ± 1	37 ± 2	40 ± 6
19	110 (0)	60 (0)	50 (0)	34 ± 2	31 ± 3	41 ± 2
20	110 (0)	60 (0)	50 (0)	34 ± 2	38 ± 1	36.5 ± 0.5

*t*: time; *T*: temperature; *S*: solvent concentration (ethanol–water, propylene glycol–water, and glycerol–water mixtures); tr: traces.

**Table 2:** Parametric coefficients, statistical information of the model fitting procedure, and optimal processing conditions in natural values that lead to optimal response values of aloesin for each alcohol–water system.

Coefficients ^#^		EtOH-W System	PG-W System	Gly-W System
Intercept	*b*_0_	33.9 ± 0.4	33.0 ± 0.8	40 ± 1
Linear terms	*b*_1_	−2.9 ± 0.4	−9.0 ± 0.9	−3 ± 1
	*b*_2_	−1.3 ± 0.6 *	−1.1 ± 0.9 *	−1 ± 1 *
	*b*_3_	−14.1 ± 0.4	−8.2 ± 0.9	−6 ± 1
Quadratic terms	*b*_11_	−2.0 ± 0.3	5.6 ± 0.6	2.5 ± 0.8
	*b*_22_	−7.2 ± 0.3	ns	5.7 ± 0.8
	*b*_33_	−3.7 ± 0.3	ns	ns
Cubic terms	*b*_222_	0.9 ± 0.3	ns	ns
Interaction terms	*b*_12_	1.1 ± 0.4	−0.8 ± 0.3 *	0.9 ± 0.4 *
	*b*_13_	1.1 ± 0.4	1.8 ± 0.8	0.9 ± 0.4 *
	*b*_23_	1.2 ± 0.4	−3.9 ± 0.8	ns
	*b*_113_	3.9 ± 0.6	5 ± 1	−4 ± 1
	*b*_122_	2.9 ± 0.6	7 ± 1	4 ± 1
	*b*_1122_	ns	−12 ± 1	−11 ± 1
**Statistical Data**	Model F-value	232.64	29.53	23.54
	Lack of Fit	ns	ns	ns
	R^2^	0.9904	0.9291	0.9099
	R^2^_adj_	0.9862	0.8976	0.8699
	Ad. Precision	57.21	24.32	17.3697
	C.V. (%)	5.80	9.85	9.32
**Optimal Conditions**	*X*_1_ (min)	92.9	12.0	42.2
	*X*_2_ (°C)	55.9	60.6	56.8
	*X*_3_ (%, *w*/*w*)	0.0	51.1	17.5
**Response Optimum**	Model-predicted	48 ± 1 mg/L	63 ± 2 mg/L	57 ± 2 mg/L
	Experimental	51 ± 4 mg/L	65 ± 4 mg/L	61 ± 3 mg/L

^#^ Parametric subscripts 1, 2 and 3 correspond to the variables *X*_1_ (time), *X*_2_ (temperature) and *X*_3_ (solvent concentration), respectively. R^2^: coefficient of determination; R^2^_ajd_: adjusted coefficient of determination; Ad. Precision: adequate precision; C.V.: coefficient of variation; ns: not significant. * Statistically non-significant (*p*-value > 0.05) terms required to maintain hierarchy.

**Table 3:** Antioxidant, antibacterial and antifungal activities of the *Aloe vera* rind extracts obtained under the optimal conditions of the studied alcohol–water systems.

	EtOH-W Extract		PG-W Extract		Gly-W Extract		Positive Control *	
**TBARS** (EC_50_, µg/mL) ^#^	310 ± 21 ^a^		432 ± 18 ^b^		610 ± 13 ^c^		5.4 ± 0.3	
**Antibacterial Activity**	MIC	MBC	MIC	MBC	MIC	MBC	MIC	MBC
*Staphylococcus aureus*	1.5	3	1.5	2	2	4	0.006	0.012
*Staphylococcus epidermidis*	1.5	2	1.5	2	na	na	0.003	0.006
*Staphylococcus lugdunensis*	2	3	1	2	2	4	0.025	0.05
*Micrococcus flavus*	2	4	1.5	2	na	na	0.2	0.3
*Listeria monocytogenes*	2	4	0.25	0.5	na	na	0.2	0.3
*Escherichia coli*	3	4	1	2	1.5	2	0.006	0.012
*Pseudomonas aeruginosa*	na	na	0.5	1	1.5	2	0.025	0.05
*Salmonella enterica* serovar Typhimurium	2	3	0.25	0.5	na	na	0.25	0.5
**Antifungal Activity**	MIC	MBC	MIC	MBC	MIC	MBC	MIC	MBC
*Aspergillus flavus*	1.5	2	1	2	na	na	0.25	0.5
*Aspergillus niger*	2	4	1.5	2	na	na	0.2	0.5
*Penicillium funiculosum*	1	2	1	2	na	na	0.2	0.5
*Candida albicans*	2	4	0.7	1	2	4	0.4	0.8
*Trichophyton mentagrophytes*	1	2	0.12	1	2	4	0.012	0.025
*Trichophyton tonsurans*	0.5	1	0.25	0.5	2	4	0.0015	0.003
*Microsporum gypseum*	1	2	0.5	1	1	2	0.006	0.012
*Microsporum canis*	1	2	1	2	0.5	1	0.003	0.006

* Trolox, streptomycin, and ketoconazole were the positive controls used in the antioxidant, antibacterial, and antifungal activity assays, respectively. ^#^ In the TBARS assay, for each solvent system, a blank was prepared with the respective extraction solvent described in Table 2. ^a–c^ Different superscript letters indicate significant differences (*p* < 0.05) between extracts. MIC: minimum inhibitory concentration (mg/mL); MBC: minimum bactericidal concentration (mg/mL); MFC: minimum fungicidal concentration (mg/mL); na: not activity.

## Data Availability

Not applicable.

## Supplementary Materials

The following are available online at https://www.mdpi.com/article/10.3390/biology10100951/s1, Figure S1: 2D response graphs for the effects of the independent variables on the aloesin content obtained from *Aloe vera* rind. In each plot, the excluded variables were positioned at their optimal value (Table 2).
