# Supplementary material for: Extraction of Aloesin from Aloe vera Rind Using Alternative Green Solvents: Process Optimization and Biological Activity Assessment
Source: Biology (Basel). 2021 Sep 23;10(10):951. doi: 10.3390/biology10100951 (PMC8533118; doi:10.3390/biology10100951)
Supplement: Supplementary file 1 [file biology-10-00951-s001.zip › biology-1380407-supplementary.pdf]

biology

MDPI

Supplementary Material

Extraction of Aloesin from Aloe vera Rind using Alternative Green Solvents: Process Optimization and Biological Activity Assessment

Mikel Añibarro-Ortega, José Pinela, Ana Ćirić, Elsa Lopes, Adriana K. Molina, Ricardo C. Calhelha, Marina Soković, Olga Ferreira, Isabel C.F.R. Ferreira, Lillian Barros

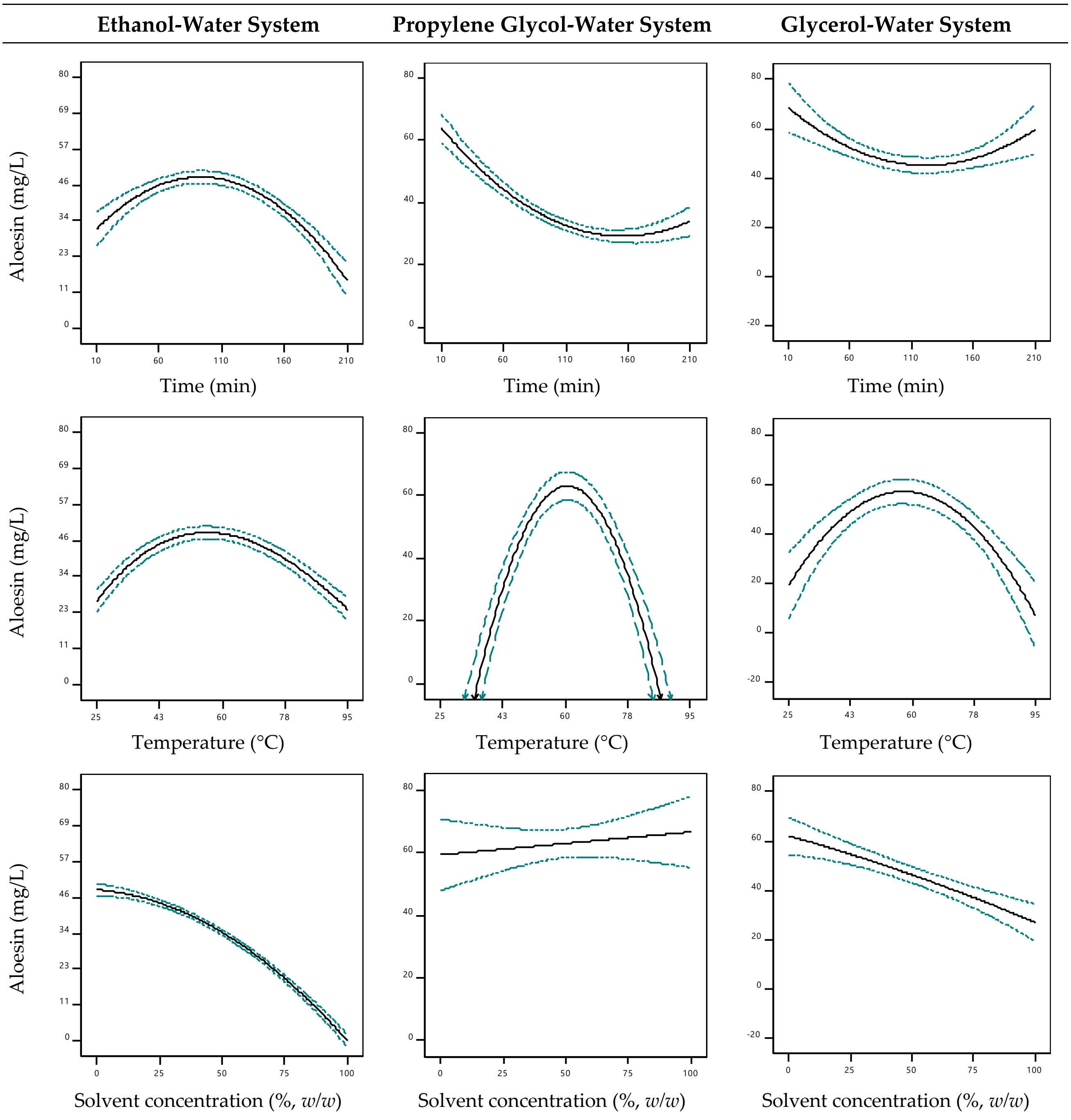

Figure S1. 2D response graphs for the effects of the independent variables on the aloesin content obtained from *Aloe vera* rind. In each plot, the excluded variables were positioned at their optimal value (Table 2).
